# Exploring miRNAs involved in blue/UV-A light response in *Brassica rapa* reveals special regulatory mode during seedling development

**DOI:** 10.1186/s12870-016-0799-z

**Published:** 2016-05-10

**Authors:** Bo Zhou, Pengzhen Fan, Yuhua Li, Haifang Yan, Qijiang Xu

**Affiliations:** College of Life Science, Northeast Forestry University, 26 Hexing Road, Harbin, 150040 China; State Key Laboratory of Tree Genetics and Breeding, Northeast Forestry University, 26 Hexing Road, Harbin, 150040 China

**Keywords:** *Brassica rapa* subsp. *rapa*, High-throughput sequencing, Light response, microRNA, cv. Tsuda, Turnip, Anthocyanin biosynthesis

## Abstract

**Background:**

Growth, development, and pigment synthesis in *Brassica rapa* subsp. *rapa* cv. Tsuda, a popular vegetable crop, are influenced by light. Although microRNAs (miRNAs) have vital roles in the metabolic processes and abiotic stress responses of plants, whether miRNAs play a role in anthocyanin biosynthesis and development of Tsuda seedlings exposed to light is unknown.

**Results:**

Seventeen conserved and 226 novel miRNAs differed at least 2-fold in response to blue and UV-A light compared with levels after a dark treatment. Real time PCR showed that BrmiR159, BrmiRC0191, BrmiRC0460, BrmiRC0323, BrmiRC0418, BrmiRC0005 were blue light-induced and northern blot revealed that the transcription level of BrmiR167 did not differ significantly among seedlings treated with dark, blue or UV-light. BrmiR156 and BrmiR157 were present in the greatest amount (number of reads) and among their 8 putative targets in the SPL gene family, only SPL9 (Bra004674) and SPL15 (Bra003305) increased in expression after blue or UV-A exposure. In addition, miR157-guided cleavage of target SPL9 mRNAs (Bra004674, Bra016891) and SPL15 mRNAs (Bra003305, Bra014599) took place 10 or 11 bases from the 5′ ends of the binding region in the miR157 sequence.

**Conclusions:**

A set of miRNAs and their targets involved in the regulation of the light-induced photomorphogenic phenotype in seedlings of *Brassica rapa* was identified, providing new insights into blue and UV-A light-responsive miRNAs in seedlings of Tsuda and evidence of multiple targets for the miRNAs and their diverse roles in plant development.

**Electronic supplementary material:**

The online version of this article (doi:10.1186/s12870-016-0799-z) contains supplementary material, which is available to authorized users.

## Background

MicroRNAs (miRNAs) are noncoding, regulatory RNAs approximately 21 nt long that negatively regulate gene expression at the transcriptional and post-transcriptional levels via post-transcriptional cleavage of mRNA, inhibition of translation and RNA-dependent RNA polymerase (RdRP)-mediated second-strand synthesis, and *trans*-acting small interfering RNAs (ta-siRNAs) initiated by miRNA and miRNA-dependent DNA methylation [1–4]. Initially discovered in *C. elegans* [5], they have now been widely reported in plants and animals [6, 7], and some miRNAs or miRNA-like RNAs have even been reported in viruses and fungi [8, 9]. In recent years, increasing evidence has demonstrated that miRNAs are involved in the regulation of many biological and metabolic processes such as development, signal transduction, metabolism and response to environmental signals in plants [10–12].

Environmental signals such as ultraviolet (UV) and blue regions of the light spectrum are important for plants to control a wide range of processes during growth and development [13, 14]. At least two classes of photoreceptors, mainly UV-B receptors and cryptochromes, absorb UV and blue light, respectively, in plants [15–18]. Blue and UV-A light-mediated photomorphogenic responses include de-etiolation, phototropism, stomatal opening, and anthocyanin accumulation [19], which are controlled by light-responsive genes. In particular, blue and UV-A light induce transcription of the genes involved in anthocyanin biosynthesis of *Brassica rapa* subsp. *rapa* [20]. Blue and UV-B light have also been reported to mediate miRNAs that regulate genes in maize and *Arabidopsis* [21, 22]. For example, in *Arabidopsis*, Cryptochrome 1 (CRY1) and Cryptochrome 2 (CRY2) mediate the expression of miR172 after blue light stimulation in a CONSTANS (CO)-independent manner to regulate photoperiodic flowering time [22]. Four miRNAs (miR160, miR165/166, miR167 and miR393) also respond to UV-B and might be involved in auxin signaling pathways of *Arabidopsis* [23]. Similarly, miRNA396 exposed to UV-B radiation in leaves of *Arabidopsis* is upregulated, leading to a decrease in transcripts of *GROWTH-REGULATING FACTOR* genes (*GRFs*) which result in inhibition of cell proliferation and leaf growth [24, 25]. Moreover, miR408 is coordinately regulated by SQUAMOSA PROMOTER BINDING PROTEIN-LIKE7 (SBP/SPL7) and ELONGATED HYPOCOTYL5 (HY5) in *Arabidopsis*, and the transcription levels of miR408 and its target genes are also changed in response to light and copper [26]. In maize, miR164, miR165, miR166, and miR398 are up-regulated, while miR156, miR171, miR172, miR396, and miR529 are down-regulated under UV-B radiation treatment. Furthermore, both miR156 and miR529 are decreased and their several targets (SBP transcripts) are increased after 8h UV-B in maize leaves [21].

To date, high-throughput sequencing has revealed novel microRNAs in different organs at different stages of development of *B. rapa* subsp. *pekinensis* cv. Chiifu [27], in seedlings of *B. rapa* subsp. *chinensis* stressed by heat [28] and in skotomorphogenic seedlings of *B. rapa* subsp*. rapa* cv. Tsuda [29]. In addition, many light signal transduction factors and genes involved in light-induced anthocyanin biosynthesis have been extensively studied in *Brassica rapa* [20, 23], but the miRNAs that are responsive to blue and UV-A light have not yet been systematically identified and characterized at the genome level.

Turnip (*Brassica rapa* subsp. *rapa*; Brassicaceae) is an important and popular cruciferous root vegetable. The turnip cv. Tsuda, a purple top cultivar, accumulates the anthocyanidin pelargonidin in swollen hypocotyls and in the upper mid-section of hypocotyls of the seedling after exposure to sunlight and blue and UV-A light [20, 30]. Because of the health-promoting role of anthocyanins, this red turnip cultivar Tsuda is very desirable, and its blue and UV-A light-induced anthocyanin biosynthesis is especially suitable to study the role of miRNAs in regulating anthocyanin production in response to various wavelengths of light.

To identify miRNAs involved in light-induced anthocyanin biosynthesis in *Brassica rapa*, high-throughput sequencing technology was used to obtain differentially expressed conserved and novel miRNAs in response to blue light, UV-A and dark treatment. A set of miRNAs and their targets were found to be involved in the regulation of light-induced photomorphogenic phenotype in seedlings of *Brassica rapa*. The most abundant miRNA156/157 could negatively regulate the transcript level of their targets SPL9 and SPL15 in the anthocyanin biosynthetic seedlings under blue light and UV-A induction. The identification of blue and UV-A light-responsive miRNAs could aid in understanding the mechanisms underlying plant response to light-induced anthocyanin biosynthesis.

## Results

### High-throughput analysis of small RNAs responsive to blue and UV-A light

**Fig. 1:**
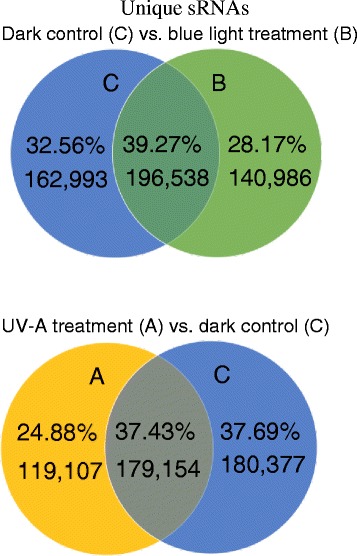
Venn diagram of unique sRNAs in sRNA library for Tsuda seedlings after blue light or UV-A treatment in comparison with the dark treatment. Left, dark-control specific sRNAs (C) vs. blue-light specific (B); right, dark-control specific sRNAs vs. UV-A specific (A)

A small RNA (sRNA) library obtained from seedlings of turnip grown in the dark for 4 days as control and induced by blue light or UV-A for 1 day after growing in the dark for 3 days as treatment were sequenced using the Solexa system. A total of 16,859,441, 16,110,664 and 13,974,772 clean reads were obtained from the dark, blue and UV-A small RNA library, respectively. Among the clean reads, small RNA sequences varied widely in length (from 10 to 44 nt); the number of 20–24-nt sequences significantly outnumbered the shorter or longer sequences (Additional file 1: Figure S1). The number of these 20–24-nt small RNAs accounted for 84.37 % of the total sequences for the dark treatment, 69.42 % for blue and 66.77 % for UV-A. The blue and the UV-A light treatments generated 337,524 and 298,261 unique sRNAs, respectively, fewer than in the dark treatment (359,531 unique sRNAs). Compared with sRNAs in the dark treatment, 28.17 % of the sRNAs were specific to blue light, while 24.88 % of the sRNAs were specific to UV-A (Fig. 1). Moreover, 63.51 %, 62.13 % and 56.75 % unique sequences were obtained from dark-, blue- and UV-A-treated seedlings, respectively, and they mapped completely onto the *Brassica* A genome from *B. rapa* (Chiifu-401) (http://brassicadb.org/brad/). Also, 7,033,801 (52.81 %), 5,365,249 (42.15 %) and 4,042,699 (36.57 %) small RNA reads from the dark, blue and UV-A small RNA library, respectively, were annotated as miRNAs. The rest of the sequences were found to be other types of RNA, including unannotated RNA, tRNA, rRNA, small nuclear RNA (snRNA), small nucleolar RNA (snoRNA) and intron or exon sequences. The numbers and proportions of different categories of small RNAs are shown in Table 1. The results revealed that the proportion of known miRNAs in blue- or UV-A-treated seedlings was lower than in the dark-grown seedlings.

**Table 1:** Distribution of small RNA reads among different categories in the sRNA library after dark, Blue light or UV-A treatment of *Brassica rapa* subsup. rapa cv. Tsuda

Category	Dark treatment				Blue light treatment				UV-A light treatment			
	Unique sRNAs	Percent (%)	Total sRNAs	Percent (%)	Unique sRNAs	Percent (%)	Total sRNAs	Percent (%)	Unique sRNAs	Percent (%)	Total sRNAs	Percent (%)
Total	359,531	100 %	13,319,035	100 %	337,524	100%	12,727,958	100 %	298,261	100 %	1,105,3984	100 %
exon_ antisense	7952	2.21 %	151,963	1.14 %	7847	2.32%	117,444	0.92 %	7742	2.60 %	167,459	1.51 %
exon_ sense	7916	2.20 %	94,268	0.71 %	7545	2.24%	100,660	0.79 %	7259	2.43 %	79,121	0.72 %
intron_ antisense	12,312	3.42 %	183,025	1.37 %	11,345	3.36%	148,510	1.17 %	9167	3.07 %	146,973	1.33 %
intron_ sense	19,413	5.40 %	274,427	2.06 %	17,941	5.32%	250,571	1.97 %	13,782	4.62 %	183,113	1.66 %
miRNA	2,349	0.65 %	7,033,801	52.81 %	2,365	0.70%	5,365,249	42.15 %	2,395	0.80 %	4,042,699	36.57 %
rRNA	20,611	5.73 %	1,235,211	9.27 %	22,614	6.70%	864,612	6.79 %	35,121	11.78 %	1,412,822	12.78 %
snRNA	311	0.09 %	2,640	0.02 %	383	0.11%	3,134	0.02 %	394	0.13 %	3,668	0.03 %
snoRNA	189	0.05 %	1546	0.01 %	457	0.14%	7999	0.06 %	324	0.11 %	3431	0.03 %
tRNA	5715	1.59 %	1,137,537	8.54 %	10,857	3.22%	2,791,679	21.93 %	11,743	3.94 %	1,945,982	17.60 %
unann	282,763	78.65 %	3,204,617	24.06 %	256,170	75.90%	3,078,100	24.18 %	210,334	70.52 %	3,068,716	27.76 %

### Identification of conserved and novel candidate miRNAs in seedlings of *B. rapa* subsp. *rapa* cv. Tsuda after different light treatments

All unique small RNA sequences were aligned with the currently known miRNAs in the miRNA database miRBase (Release 20) and then screened to determine their hairpin structures. In total, 138 precursors belonging to 54 conserved miRNA families in the dark-responsive miRNAs, 140 precursors belonging to 54 conserved miRNA families in the UV-A-responsive miRNA library, and 129 precursors belonging to 53 conserved miRNA families in the blue-light-responsive miRNA library were separately identified as homologs of known miRNA families from diverse plant species (Additional file 2: Table S1). Among these families, miR156, miR157, miR167, miR168 were abundant, accounting for more than 90 % of the total conserved and novel miRNA reads in dark-, blue-light- and UV-A-responsive sRNA libraries.

To uncover novel miRNAs from *B. rapa*, all unannotated sRNAs were analyzed using a bioinformatic approach. According to criteria for annotating novel plant miRNAs [31], 70 miRNA sequences (including their miRNA^*^s) from 126 loci were identified (Additional file 3: Table S2). Among these miRNAs, 10 novel miRNAs (BrmiRC0035, BrmiRC0091, BrmiRC0099, BrmiRC0144, BrmiRC0153, BrmiRC0191, BrmiRC0211, BrmiRC0250, BrmiRC0415, BrmiRC0460) were found in all three sRNA libraries. Only four novel miRNAs (BrmiRC0311, BrmiRC784, BrmiRC799, BrmiRC839) were detected in blue-light- and UV-A-specific-responsive sRNA libraries.

### Differential expression of miRNAs in response to dark, blue and UV-A light

**Fig. 2:**
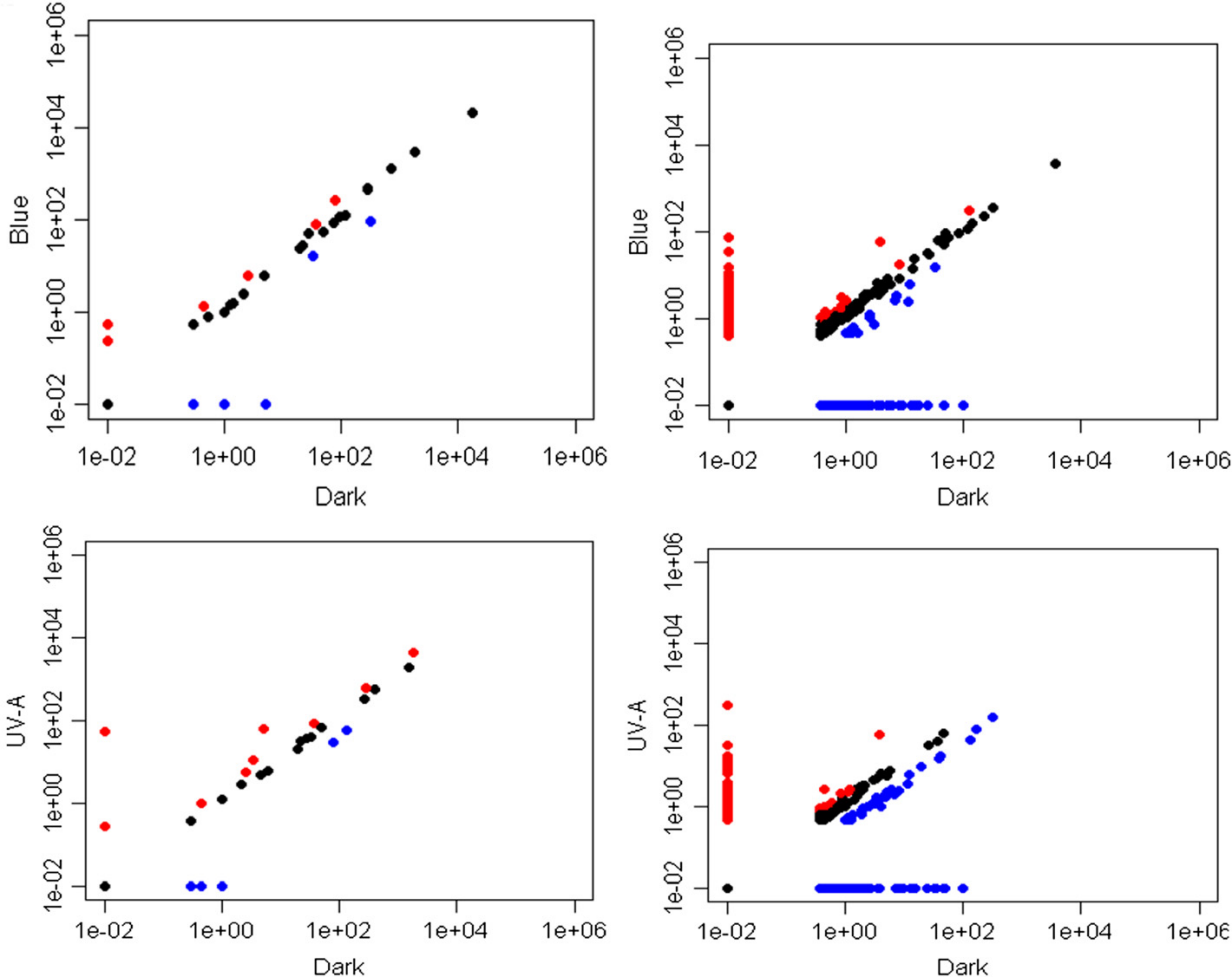
Differential expression of miRNAs in Tsuda seedings after dark, blue light or UV-A treatment. Red dots denote the ratio of log 2(*y*_treatment_/*x*_control_) ≥ 1; blue dots denote the ratio of log 2 (*y*_treatment_/*x*_control_) ≤ −1

Based on the results of high-throughput sequencing, miR5175 and miR3630 were only detected in the UV-A sRNA library, miR5300 was only found in the blue sRNA library, and miR1535 and miR5138 were identified only in the dark sRNA library. By normalizing the number of miRNA reads (on the basis of transcripts per million, TPM) in the library, the relative abundance of miR391, miR1439, miR2111, miR2911, miR2916, miR5083 in the UV-A sRNA library was 2 times higher than in the dark sRNA library, whereas the abundance of miR396 and miR1885 was the opposite. In the blue-light-responsive sRNA library, aside from miR391, miR2111 and miR5083, miR1511 was more abundant than in the dark sRNA library, whereas miR5072 and miR5139 were less abundant. However, the transcription level of most miRNAs did not obviously differ in the seedlings among the dark, blue light and UV-A light treatments (Additional file 4: Table S3). Among the novel miRNAs, 23 candidate miRNAs were downregulated, and 7 were upregulated more than 2-fold after UV-A treatment. In particular, BrmiRC0305 and BrmiRC0491 were upregulated more than 4-fold after UV-A. Moreover, 9 candidate miRNAs were downregulated, and 10 candidate miRNAs were upregulated more than 2-fold after blue light. There were also 110 candidate miRNAs specific to the UV-A-light-induced library, 67 candidate miRNAs specific to blue-light-induced library and 226 specific to the dark-treatment library (Fig. 2).

### Expression analysis of potential miRNAs responsive to dark, blue and UV-A light by qRT-PCR and RNA blot

**Fig. 3:**
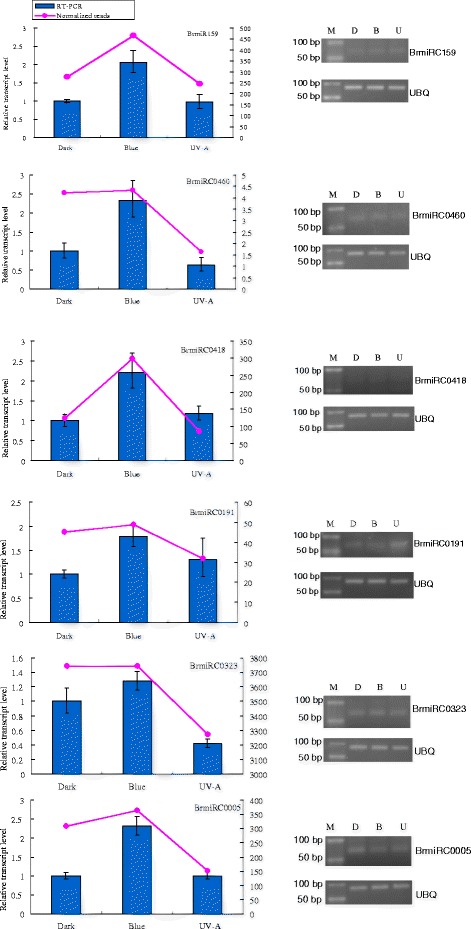
Transcript expression of conserved and novel miRNA predicted from Tsuda after treatment with dark, blue light or UV-A. Relative expression (2^−ΔΔCT^) of conserved BrmiR159 and predicted BrmiRC0191, BrmiRC0460, BrmiRC0323, BrmiRC0418, BrmiRC0005 was analyzed by real time PCR, and the qRT-PCR products were separated by electrophoresis. The UBQ gene was used as the internal control, and seedlings in the dark control were used as the calibrator. Lanes: M, size marker (10 bp DNA Ladder, MBI); D, dark; B, blue light; A, UV-A. Each bar indicates the mean ± SE of triplicate assays after correcting for template quantity relative to the UBQ gene. The dotted line indicates the normalized reads for the corresponding miRNAs from high-throughput sequencing

**Fig. 4:**
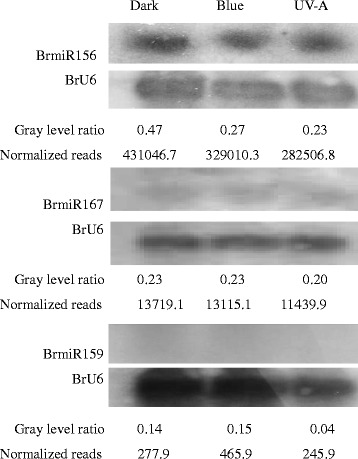
Northern blot analysis of BrmiR156, BrmiR167 and BrmiR159 in seedlings of Tsuda after exposure to dark, blue light or UV-A. 5′-Digoxigenin-labeled DNA oligonucleotide with complementary sequence to miRNA was used as the probe; BrU6 was used as the control. The gray level ratio between miRNAs and BrU6 was used to compare relative expression to normalized reads

To confirm the original high-throughput sequencing results, we selected three conserved miRNAs (BrmiR156 with more than 3 million reads, BrmiR167 with more than 100,000 reads, and BrmiR159 with more than 2000 reads) as examples to analyze their expression using an RNA blot. In addition, five novel candidate miRNAs and BrmiR159 were analyzed by real time PCR. The RNA blot and real time PCR results demonstrated that all tested miRNAs were expressed in seedlings in the dark, blue light and UV-A treatments and that the transcription levels of the different miRNAs varied (Figs. 3, 4). Transcription of most of the selected miRNAs (BrmiR159, BrmiRC0191, BrmiRC0460, BrmiRC0323, BrmiRC0418, BrmiRC0005) was higher after the blue light treatment than after the dark or the UV-A light treatment (Fig. 3). Northern blot showed that the transcription level of BrmiR167 was not significantly different among the seedlings from the dark, blue and UV-light induction, while the expression of BrmiR156 was slightly inhibited by blue and UV-A light (Fig. 4). When the relative expression levels of candidate microRNAs shown in RNA blots (gray level ratios between the blot results of microRNAs and the U6) or by RT-PCR were compared with the normalized reads of microRNAs from the high-throughput sequencing library of the dark, blue light and UV-A light induced seedlings, all selected miRNAs had the same expression profiles as in the original high-throughput sequencing results (Fig. 3, 4).

### Prediction of miRNA target genes

The putative target genes for all identified miRNAs from the seedlings in the different light treatments were predicted by using psRNA Target program. In total, 270 potential targets genes were found for 51 conserved miRNAs and 3589 for 802 novel miRNAs (Additional file 5: Table S4). Many of the miRNAs had multiple targets, indicating the diverse regulatory roles of these miRNAs. Certainly, the targets that are common to more than one miRNA, such as Bra026884 (target of miR161 and miR400), Bra000791 (target of BrmiRC0930 and BrmiRC0965), Bra000965 (target of BrmiRC0598 and BrmiRC0776), Bra001160 (target of BrmiRC0149 and BrmiRC0801), also have different miRNA-binding sites, indicative of the complex regulatory network of miRNAs. Most of these putative target genes encode transcription factors such as SPL (targets of miR156, miR157 and miR1088), Auxin response factor (ARF) (targets of miR160 and miR167), Myeloblastosis (MYB) (targets of miR159 and miR828), Basic leucine zipper (bZIP) (BrmiRC0651, and BrmiRC0940), AGAMOUS-like (AGL) (targets of miR824 and BrmiRC0607), WRKY (targets of BrmiRC0049, BrmiRC0149, BrmiRC0177 and BrmiRC0181) and APETALA2 (AP2) (targets of miR172), which have known or putative functions in a wide variety of biological processes. Interestingly, miR164, BrmiRC0117, BrmiRC0178, BrmiRC0601, BrmiRC0982 targeted genes that encode NAM, ATAF1, 2, and CUC2 (NAC) proteins, and miR1885, BrmiRC0149, BrmiRC0177, BrmiRC0311, BrmiRC0801, BrmiRC0883 targeted genes encoding a disease resistance protein ((Toll Interleukin-1 Receptor) TIR- (Nucleotide-Binding Site) NBS- (Leucine-Rich Repeat) LRR class). Genes targeted by miR161, miR400 and BrmiRC0797 encode a pentatricopeptide (PPR) repeat-containing protein. In addition, some miRNAs targeted genes that are involved in light signal transduction and morphogenic responses. For example, miR2111 was predicted to target a cop8 (constitutive photomorphogenic) homolog, miR399 was predicted to target SPA2 (SPA1-RELATED 2), BrmiRC0406 was predicted to target CRY2, and BrmiRC0826 was predicted to target phytochrome and flowering time regulatory protein 1.

### Analysis of miR156, miR157 and their targets after blue light and UV-A exposure

In the high-throughput sequencing analyses of the blue light and UV-A induction compared with the dark treatment, miR156 and miR157 had about 1.5-fold change in their transcript numbers. RT-PCR also showed a significant change in expression of miR157 after UV-A treatment compared with the dark treatment (two-tailed *t*-test, *p*-value is 0.0259, t = 3.132, df = 5), but under blue light treatment compared with the dark treatment the expression did not differ significantly (*P <* 0.05) (p-value is 0.129, t = 1.817, df = 5) (Additional file 6: Table S5).

**Fig. 5:**
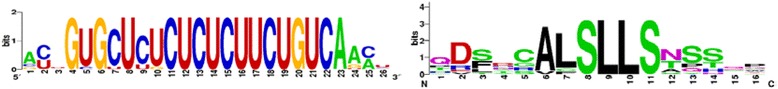
Conserved sequence analysis of predicted binding site for miR156 and miR157 in target mRNA and amino sequence. Multiple sequence alignment was done using WebLogo (http://weblogo.berkeley.edu/logo.cgi)

**Fig. 6:**
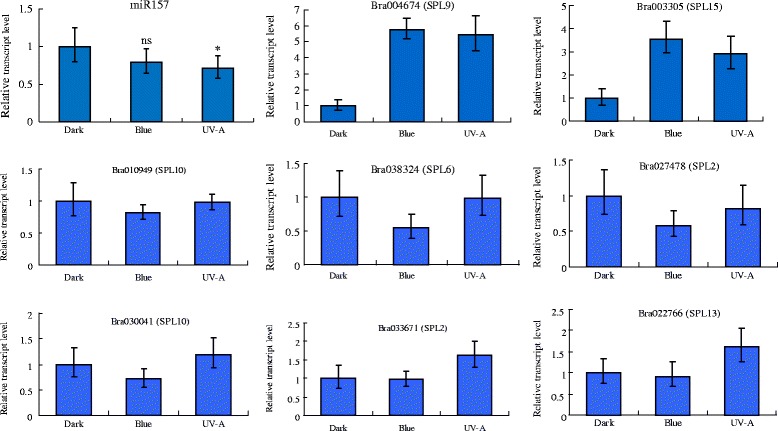
Mean relative transcript level of BrmiR157 and its potential target BrSPL genes in seedlings of Tsuda exposed to dark, blue light or UV-A. Relative expression (2^−ΔΔCT^) of conserved BrmiR157 and predicted Bra004674 (SPL9), Bra003305 (SPL15), Bra010949 (SPL10), Bra038324 (SPL6), Bra027478 (SPL2), Bra030041 (SPL10), Bra033671 (SPL2), Bra022766 (SPL13) were analyzed by real time PCR. The UBQ gene served as the internal control to correct for template quantity; seedlings in the dark control served as the calibrator. Bar indicates ± SE of triplicate assays. Statistically significant differences between blue light treatment and dark, UV-A treatment and dark are shown (*t*-test, *P < 0.05; ns, not significant difference)

**Fig. 7:**
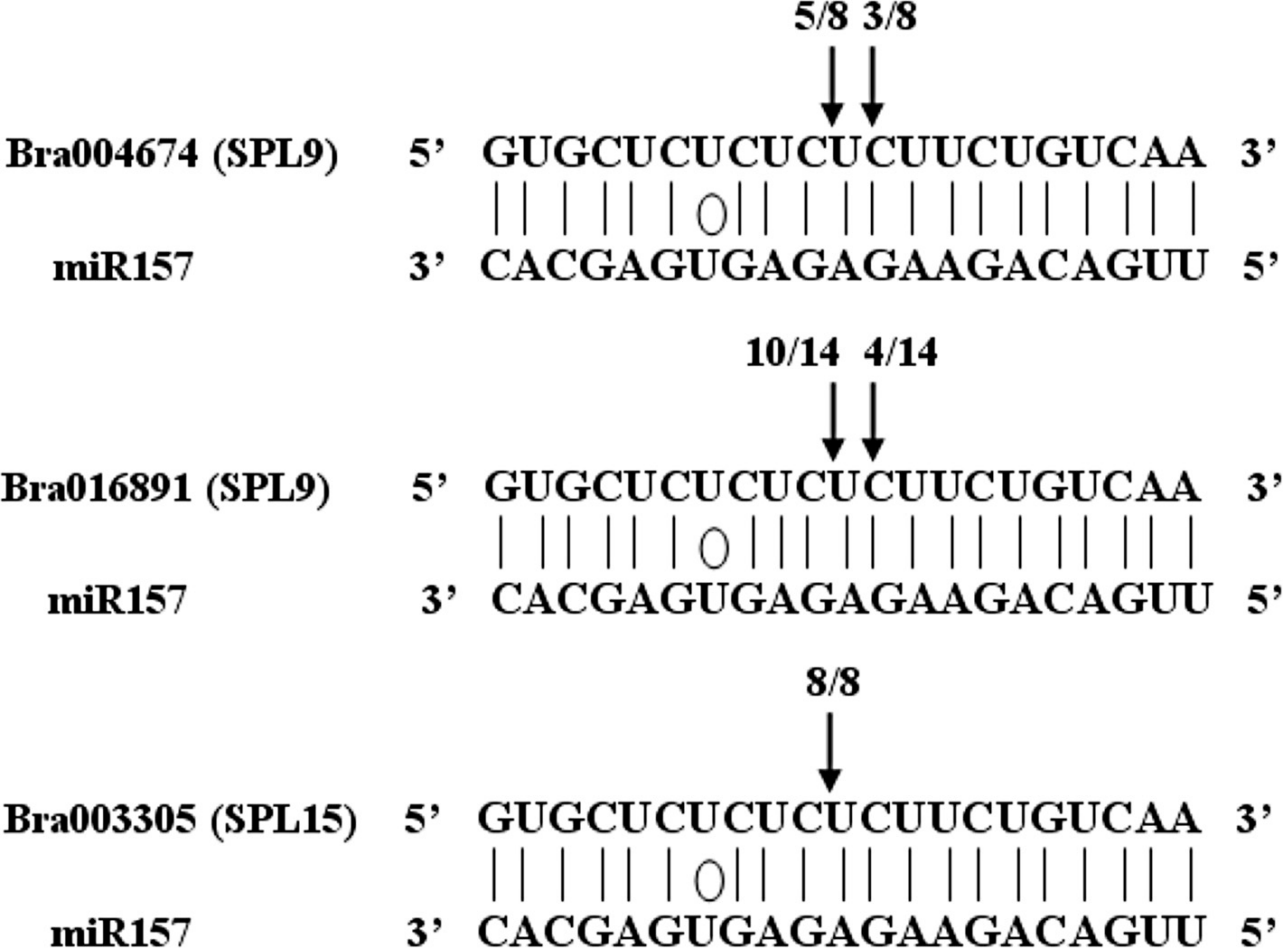
Sites of BrmiR157-mediated cleavage on target BrSPL9 and BrSPL15 mRNAs. Positions of dominant 5′-RACE products of BrSPL9 (Bra004674, Bra016891) and BrSPL15 (Bra003305) mRNA are indicated by a vertical arrow in the binding region

We then explored miR156 and miR157 and their putative targets in more detail. The 13 target genes of miR156 and miR157 belong to 7 members of the *SPL* gene family and have highly conserved sequences in the binding site (Table 2 and Fig. 5). RT-PCR showed that the expression of predicted targets *SPL9* (Bra004674) and *SPL15* (Bra003305) induced by blue light and UV-A was approximately 3 times higher than in the dark treatment. The transcript level of the other detected targets, *SPL10* (Bra010949 and Bra030041), *SPL2* (Bra027478 and Bra033671), *SPL6* (Bra038324) and *SPL13* (Bra022766), did not differ significantly among seedlings grown in the dark, blue or UV-A light (Fig. 6). The miR157-guided cleavage of target *SPL9* mRNAs (Bra004674, Bra016891) and *SPL15* mRNAs (Bra003305, Bra014599) was detected as expected. The cleavage sites were 10 or 11 bases from the 5′ ends of the binding region in the miR157 sequences (Fig. 7) (Additional file 7: Figure S2).

**Table 2:** Predicted binding site of miR156 and miR157 on sequences of candidate target genes in SPL family

Candidate gene	Start region number in mRNA sequence	mRNA sequence of predicted binding site area	Amino acid sequence of corresponding binding site area	Stop region number in amino sequence
Bra027478 (SPL2)	922	GAUGGUGCUCUCUCUCUUCUGUCAAAU	QDLDGALSLLSNSTAW	316
Bra033671 (SPL2)	841	GAUGGUGCUCUCUCUCUUCUGUCAAAU	QDLDGALSLLSNSTPW	293
Bra004363 (SPL6)	631	ACUUGUGCUUGCUCUCUUCUGUCAGCU	PRSTCACSLLSAQSQQ	223
Bra038324 (SPL6)	673	ACUUGUGCUCUCUCUCUUCUGUCAGCU	PRSTCALSLLSAQSQQ	237
Bra016891 (SPL9)	736	AACUGUGCUCUCUCUCUUCUGUCAAAU	GDSNCALSLLSNPHQ	257
Bra004674 (SPL9)	739	AACUGUGCUCUCUCUCUUCUGUCAAAC	SVTNCALSLLSNPHQP	259
Bra010949 (SPL10)	991	UACAGUGCUCUCUCUCUUCUGUCAACG	QDFYSALSLLSTSSDS	343
Bra030041 (SPL10)	1015	UACAGUGCUCUCUCUCUUCUGUCAACG	QDFYSALSLLSTSSDS	351
Bra030040 (SPL11)	1067	CACCGUGCUCUCUCUCUUCUGUCAACU	QEFHRALSLLSTSSSD	369
Bra032822 (SPL11)	994	CACCGUGCUUUCUCUCUUCUGUCAACU	QDFHRAFSLLSTSSGP	344
Bra022766 (SPL13)	844	GAUUGCGCUCUCUCUCUUCUGUCAUCA	HDSDCALSLLSSSSSH	294
Bra003305 (SPL15)	562	AGCUGUGCUCUCUCUCUUCUGUCAAAC	TDSSCALSLLSNYTNP	201
Bra014599 (SPL15)	550	AGCUGUGUUCUCUCUCUUCUGUCAAAC	TDSSCVLSLLSNSNTT	196

## Discussion

In this study, high-throughput sequencing was used to obtain more knowledge about gene regulation by miRNAs induced by blue light and UV-A, which induce anthocyanin biosynthesis in seedlings of the Tsuda cultivar of *Brassica rapa* subsp. *rapa*. By comparing the miRNAs and the miRNA precursors in *Arabidopsis* with the sRNA library of blue- and UV-A-treated seedlings of Tsuda turnip, we were able to identify the most common conserved miRNA families in the cultivar. Certainly, many Tsuda turnip-specific miRNAs were also obtained, but the number of reads was limited (from 5 to 50,000), and most were not over 1000. This result is consistent with our previous research on the dark-treated seedlings [29].

As technology has developed, a number of miRNAs have been identified in plants, and their roles in regulation have begun to be elucidated. For example, higher levels of pigments accumulated in transgenic *Arabidopsis* which over-expressed HY5-regulated miR408, resulting in a phenotype opposite of the lower pigment levels caused by a mutation in *hy5* [32]. MiR172 mediates photoperiodic flowering independent of *CONSTANS* in *Arabidopsis* [22, 33]. High-throughput sequencing of *Brassica rapa* subsp. *chinensis* showed that miR398, miR399, miR827, miR5716 and miR1885 were downregulated under heat stress, while miR156, miR5714, miR5718 and miR5726 significantly increased [28]. In our light-induced RNA library, BrmiR391, BrmiR2111, BrmiR5083 and BrmiRC0132, BrmiRC0448, BrmiRC0491 were induced by both blue light and UV-A. Furthermore, miR156/157, miR159/319, miR160, miR165/166, miR167, miR169, miR170/171, miR172, miR393, miR398 and miR401, which are responsive to UV-B in *Arabidopsis* [34], were all detected in light-treated seedlings of *Brassica rapa*, but most had no obvious difference in transcription level. Only miR156/157, miR398 induced by UV-A, miR159, miR319 induced by blue light, miR172 induced by blue and UV-A light showed over 1.5-fold change in expression compared with the levels in dark-treated seedlings (Additional file 4: Table S3). In the analysis of the predicted targets, the BrmiR391 target was annotated as flavin adenine dinucleotide (FAD) binding, the BrmiR2111 target as a COP8 (constitutive photomorphogenic) homolog, the BrmiR5083 target as a potassium transporter family protein, the BrmiRC0132 target as a cytochrome family protein, the BrmiRC0448 target MYB domain protein 16, the BrmiRC0491 target as potassium transporter 11. Most of these targets are involved in photomorphogenesis and signal transduction [35–38].

Interestingly, miR156 and miR157 had the most reads in our detected sRNA libraries. Although the fold change in transcript levels decreased about 1.5 fold in the blue light and in UV-A treatment compared with the dark treatment, the read numbers for miR156 and miR157 were over 100,000 less in the Tsuda seedlings grown in blue or UV-A light than in the dark. Additionally, the RNA blot and the RT-PCR also showed little downregulation of miR156 or miR157 in blue light and UV-A light. The downregulation expression pattern of miR156 in light-induced anthocyanin synthesis seedlings of turnip is consistent with that in *Arabidopsis* under exogenous sugar induction [39, 40]. The level of miR156 is also reduced when the plant grows from the juvenile phase to adult phase and the expression of miR156 dwindles, ultimately leading to the reproductive phase transition [41–43]. The targets of miR156 and miR157, SPL transcription factor families, play important roles in regulating diverse aspects of plant growth and development including the vegetative phase and floral transition in *Arabidopsis* [44, 45]. Moreover, miR156 is also positively regulated by SPLs, and a negative feedback loop regulates the expression of its targets [45]. Our results also showed that the miR156 and miR157 targets, SPL9 and SPL15, were specifically upregulated by blue light and UV-A light in Tsuda seedlings. The difference of expression between miR156/157 and SPL9, SPL15 suggests that miR156 or miR157 may target SPL9 and SPL15 more effectively than other SPLs and may be involved in photomorphogenesis induced by blue light and UV-A light during the seedling development of Tsuda. A regulatory relationship may exist between miRNA 156, miRNA157 and their targets SPL9, SPL15 although we cannot exclude the possibility that SPL9 and SPL15 are upregulated by other blue or UV-A light responsive transcription factors.

**Fig. 8:**
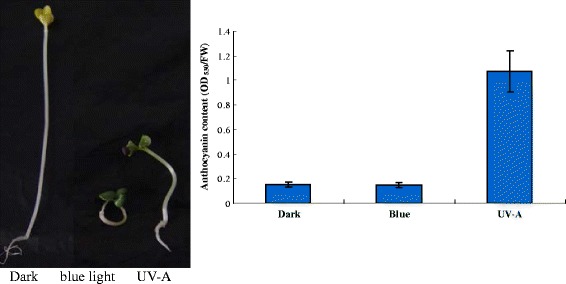
Phenotype of *Brassica rapa* subsp. *rapa* cv. Tsuda seedling and anthocyanin content after dark, blue light or UV-A treatment. Seedlings were exposed to blue light at 10 W m^−2^, UV-A at 3 W m^−2^, or to no light (dark). Anthocyanin was extracted from fresh whole seedlings, and the concentration was determined using absorbance at OD_530_ per gram of fresh mass. Vertical bars indicate ± SE (*n* = 8)

The SPL genes targeted by miR156 can be grouped into four categories: SPL9/SPL15, SPL6/SPL13, SPL11/SPL10/SPL2 and SPL3/SPL4/SPL5 [46]. SPL9 and SPL15 regulate shoot maturation and leaf initiation [47]. Overexpression of miR156 in switchgrass (*Panicum virgatum*) [48], *Arabidopsis* [49] and *Brassica napus* [50] led to reduced SPL15 expression, increased shoot branching or enhanced carotenoid content [48–50]. On the other hand, reduced miR156 activity in *Arabidopsis* results in a high level of SPL9 and negatively regulates anthocyanin accumulation by competing with TT8 in binding to anthocyanin-specific R2R3-MYBs to disrupt the stabilization of the MYB-bHLH-WD40 (MBW) complex [51]. The activity of MBW transcription factor complexes is essential to activate anthocyanin biosynthesis genes and thus for anthocyanin pigments to accumulate [52]. In this regard, the stability of the MBW complex might be increasingly impaired as the level of miR156 and miR157 transcripts decreases in seedlings of turnip exposed to blue light and UV-A light. The *Arabidopsis* mutant *sk156* expresses miR156b at a higher level than in the wild type, and miR156b-induced *SPL15* suppression is partially responsible for the increased carotenoid abundance in seeds and altered morphology of the mutant adult plant [49]. Moreover, photosynthates promote the vegetative phase change by repressing the expression of miR156 in *Arabidopsis* [39]. Our results also showed that when dark-grown seedlings were exposed to blue or UV-A light, the transcript level of miR156 and miR157 decreased, thus reducing the suppression of their targets *SPL9* and *SPL15*; the seedlings had the photomorphogenic phenotype such as anthocyanin biosynthesis, short hypocotyls, and cotyledon expansion (Fig. 8).

On the basis of our data and previous studies [20, 23, 30, 48–51], we can deduce that blue light/UV-A activates light signal transduction genes, some of which positively regulate anthocyanin biosynthesis in seedlings of *Brassica rapa* subsp. *rapa* cv. Tsuda. When positive anthocyanin biosynthesis regulators such as R2R3-MYB, basic helix-loop-helix (bHLH), WD40 [53] express increasingly by light induction, then negative feedback involving miRNAs such as miR156, miR157 and their targets SPL9 and SPL15 are also activated to negatively regulate the anthocyanin biosynthetic pathway and maintain a new balance for the transcription of the biosynthetic genes in seedlings of Tsuda turnip. Thus, light induces the maximum expression of these regulated genes. Previous studies on Tsuda seedlings also showed that the expression of chalcone synthase gene (*BrCHS*), dihydroflavonol 4-reductase gene (*BrDFR*) and Production of Anthocyanin Pigment 1 gene (*BrPAP1 or MYB75*), which are activated by MBW in blue light and UV-A, declined to low levels after they reached a peak [20].

## Conclusions

Light-responsed miRNAs were identified by Solexa sequencing of the sRNA library from photomorphogenic seedlings of *Brassica rapa* subsp. *rapa* cv. Tsuda exposed to blue light and UV-A. A qRT-PCR and northern blot analysis confirmed the expression profiles of a subset of conserved and novel miRNAs that were identified by high-throughput sequencing from the seedlings grown in the dark, blue light, or UV-A. Many putative targets for these miRNAs were predicted to be involved in plant growth, development and response to light. Transcripts of miR156 and miR157 were the most abundant in the sRNA library, and their respective targets SPL9 and SPL15 were shown to vary greatly in transcription level during seedlings photomorphogenesis from dark to blue-light- or UV-A-light-exposed. These results provide new insights into blue and UV-A light-responsive miRNAs in the seedlings of turnip and supply evidence for miR156/157-guided cleavage of target SPL9 (Bra004674, Bra016891) and SPL15 (Bra003305, Bra014599).

## Methods

### Plant materials and light treatments

Seeds of the turnip *Brassica rapa* L*.* subsp. *rapa* cv. Tsuda which originated from Dr. Kawabata Saneyuki’s laboratory (University of Tokyo) were sown in a row on wet filter paper. The seedlings were grown in the dark at 25 °C for 3 days until they were approximately 4 cm tall. For light treatments, these dark-grown seedlings were then irradiated with blue light (470 nm light-emitting diode LED, NSPB5205, Nichia) at 10 W m^−2^ or UV-A light (UV-A fluorescent lamp, FL10BLB, Toshiba, filtered through soda-lime glass plates, peak at 350 nm) at 3 W m^−2^ for 24 h. For the dark control, seedlings were grown in the dark. After a 24-h irradiation, whole seedlings (ground parts and roots) were collected and ground in liquid nitrogen to isolate total RNA using TRNzol-A^+^ reagent according to the manufacturer’s instructions (TIANGEN, Biotech, Beijing, China). Anthocyanin was estimated as described previously [23] by calculating the ratio of OD_530_ to gram fresh mass.

### Small RNA isolation and sequencing

A turnip seedling RNA library was constructed according to previously described methods [54]. In brief, small RNAs 18–30 nt long were separated from total RNA on a 15 % denaturing polyacrylamide gel and purified. The isolated small RNAs were then ligated to a 5′ adapter and a 3′ adapter and reverse transcribed into cDNA and subsequently amplified by PCR. After purification, small RNA libraries were sequenced directly using Solexa sequencing technology by Beijing Genomics Institute (BGI) (Shenzhen, Guangdong, China).

### High-throughput sequencing data analysis and miRNA identification

Clean reads were obtained by removing all low-quality reads, adapter reads, and contaminant reads. The high-quality sRNA sequences were then used to analyze length distribution and mapped to *Brassica rapa* genome sequences (http://brassicadb.org/brad/index.php) using the program SOAP [55]. Noncoding RNAs, e.g., rRNAs, tRNAs, snRNAs (small nuclear RNA) and snoRNAs (small nucleolar RNA), in the NCBI GenBank (http://www.ncbi.nlm.nih.gov/genbank/) and Rfam (http://www.sanger.ac.uk/Software/Rfam) databases were eliminated [56, 57]. The conserved miRNAs were annotated by sequence alignment using the database miRBase20.0 (http://www.mirbase.org/index.shtml) with 0- to 3-base mismatches, with gaps counted as mismatches [58]. Potential novel miRNAs were identified using Mireap (http://sourceforge.net/projects/mireap/) and mfold (http://unafold.rna.albany.edu/?q=mfold) to predict whether they can be folded into a hairpin secondary structure [59].

### Prediction of potential target genes for *Brassica rapa* miRNAs

The potential targets of conserved and novel miRNAs were predicted by the psRNA Target program (http://plantgrn.noble.org/psRNATarget/) using default parameters. *Brassica rapa*, de novo scaffolds assembly v1.1 (2011–08–30) cds, (http://brassicadb.org/brad/) was used as the genomic library for the target search. The functional annotations of these target genes were then analyzed using the Phytozome v7.0 transcript database (http://www.phytozome.net/).

### Small RNA blot analysis

Small RNA blots were analyzed as described previously [60] by probing the target small RNAs separated in a 4 % agarose gel using a 5′-digoxigenin-labeled DNA oligonucleotide probe complementary to the miRNA sequence. U6 snRNA was detected as a control and the sequence of the probe (BrU6) was 5′TTCTCGATTTGTGCGTGTCATCCTTGCGCAGGGGCCATGCTAATCTTCTC, with a digoxigenin molecule at the 5′ end. The relative expression of the detected miRNAs was calculated as the gray level ratio of images between miRNAs and BrU6.

### Reverse transcription (RT) and real time PCR analysis of miRNAs

Total RNA from turnip seedlings exposed to blue or UV-A light or kept in the dark and stem-loop primers for miRNAs and a reverse primer for the housekeeping gene were used for multiplex cDNA synthesis. [61–63]. The cDNA samples were then used for real-time PCR using miRNA-specific primers. Primers for both RT and real time PCR are listed in Additional file 8: Table S6 and Additional file 9: Table S7.

Quantitative real-time PCR was carried out on an ABI 7500 real-time system (Applied Biosystems, California, USA) with the POWER SYBR GREEN PCR Master Mix (Applied Biosystems). The RT-PCR assays for the miRNAs were done in triplicate for each cDNA sample. The PCR thermal cycling parameters were 95 °C for 10 min followed by 40 cycles of 95 °C for 15 s and 60 °C for 1 min. A comparative CT (ΔΔCT) method was used to calculate the relative amounts of the transcripts [64], then the RT-PCR products were separated by gel electrophoresis. Amplification of a fragment of the UBQ gene was chosen as an internal control, and the seedlings in the dark control were used as a calibrator. All experiments were done with three biological replicates and three technical replicates. The reaction specificities were tested with melt gradient-dissociation curves, then electrophoresis.

### 5′ RNA ligase-mediated RACE (RLM-5′RACE) PCR

Total RNA (3 μg) from light-treated seedlings was mixed equally and purified using PolyA Tract mRNA Isolation System IV (Promega, Madison, WI, USA), according to the manufacturer’s instructions. The 5′-Full RACE Kit (TaKaRa, Dalian, China) was used without the alkaline phosphatase and tobacco acid pyrophosphatase steps to detect the cleavage sites of the miRNA-targeted genes. According to the manufacturer’s instructions, the 5′ RACE adapter was directly ligated to mRNA. The nested 5′ RACE Outer/Inner primer and three gene-specific nested primers were used for rapid amplification of cDNA ends (Additional file 10: Table S8). The PCR products were purified in a 2 % agarose gel, cloned into the pCRII vector (Invitrogen, Carlsbad, CA, USA), and at least 15 independent clones were sequenced.

### Availability of data and materials

The data sets supporting the conclusions of this article are included within the article and its additional files.

### Ethics

Not applicable.

### Consent to publish

Not applicable.

### Availability of data and materials

All the supporting data are included as additional files.

## Additional files

Additional file 1: Figure S1. Length distribution and abundance of the sRNA sequences in *Brassica rapa* subsp. *rapa* cv. Tsuda seedlings after dark, blue light and UV-A light treatments. (TIF 457 kb) Additional file 2: Table S1. List of identified conserved miRNAs of Brassica rapa turnip seedings exposed in dark, blue light and UV-A. (XLS 170 kb) Additional file 3: Table S2. List of identified novel miRNAs of Brassica rapa turnip seedings exposed in dark (indicated by D), blue light (indicated by B) and UV-A (indicated by A). (XLS 93 kb) Additional file 4: Table S3. List of conserved and novel miRNAs that are differentially expressed in Brassica rapa turnip seedings after growth in dark (indicated by D), blue light (indicated by B) and UV-A (indicated by A). (XLS 288 kb) Additional file 5: Table S4. List of predicted target genes of conserved and novel miRNAs identified in Brassica rapa turnip seedings exposed to dark, blue light and UV-A. (XLS 1073 kb) Additional file 6: Table S5. Statistical analyses of real-time PCR data for miR157 (XLS 86 kb) Additional file 7: Figure S2. Identification of miR157 cleavage sites on its targets (DOCX 165 kb) Additional file 8: Table S6. Real-time RT-PCR examined miRNAs and primers. (DOC 35 kb) Additional file 9: Table S7. Real-time PCR primers for detected genes. (DOC 32 kb) Additional file 10: Table S8. Nest primers used in 5′-RACE PCR. (DOC 32 kb)

## Abbreviations

miRNAs: microRNAs
sRNAs: small RNAs
snRNA: small nuclear RNA
snoRNA: small nucleolar RNA
qRT-PCR: quantitative reverse transcription PCR
SBP/SPL: SQUAMOSA Promoter-Binding Protein-Like
UV-A: ultraviolet A
RdRP: RNA-dependent RNA polymerase
ta-siRNAs: trans-acting small interfering RNAs
CRY1: cryptochrome 1
CRY2: cryptochrome 2
CO: CONSTANS
GRFs: growth-regulating factors
COP8: constitutive Photomorphogenic 8
MYB: myeloblastosis
bHLH: basic helix-loop-helix
bZIP: Basic leucine zipper
NAC: NAM, ATAF1, 2, and CUC2
SPA2: suppressor of phya-105 (spa1)-related 2
MBW: MYB-bHLH-WD40
HY5: long hypocotyl 5
PPR: pentatricopeptide
TIR: Toll interleukin-1 receptor
NBS: nucleotide-binding site
LRR: C-terminal leucine-rich repeat
RLM-RACE: RNA ligase-mediated 5′ amplification of cDNA ends
BRAD: Brassica database
TPM: transcripts per million
UBQ: ubiquitin
RT: reverse transcription
ARF: auxin response factor
FAD: flavin adenine dinucleotide
AP2: APETALA2
AGL: AGAMOUS-like
*CHS*: chalcone synthase gene
*DFR*: dihydroflavonol 4-reductase gene
*PAP1*: Production of Anthocyanin Pigment 1 gene
