# Supplementary figures and images for: Exploring miRNAs involved in blue/UV-A light response in Brassica rapa reveals special regulatory mode during seedling development
Source: BMC Plant Biol. 2016 May 10;16:111. doi: 10.1186/s12870-016-0799-z (PMC4862165; doi:10.1186/s12870-016-0799-z)

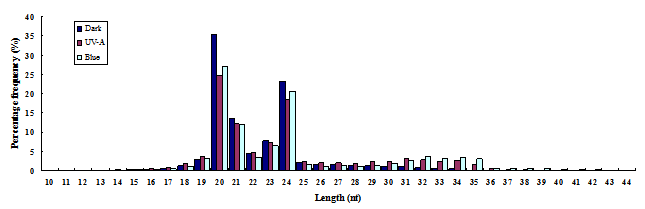

Supplement: Additional file 1: Figure S1. — Length distribution and abundance of the sRNA sequences in Brassica rapa subsp. rapa cv. Tsuda seedlings after dark, blue light and UV-A light treatments. (TIF 457 kb) [file 12870_2016_799_MOESM1_ESM.tif]
