# Supplementary material for: Exploring miRNAs involved in blue/UV-A light response in Brassica rapa reveals special regulatory mode during seedling development
Source: BMC Plant Biol. 2016 May 10;16:111. doi: 10.1186/s12870-016-0799-z (PMC4862165; doi:10.1186/s12870-016-0799-z)
Supplement: Additional file 7: Figure S2. — Identification of miR157 cleavage sites on its targets (DOCX 165 kb) [file 12870_2016_799_MOESM7_ESM.docx]

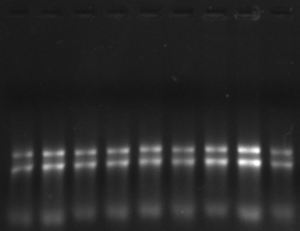


D1 D2 D3 B1 B2 B3 A1 A2 A3

Figure S2 A: Electrophoresis of total RNA from seedlings of Brassica rapa turnip under dark, blue light and UV-A treatment (D1-3, Dark treatment; B1-3, Blue light treatment; A1-3, UV-A treatment)

M 3 4

M 5 6

M 1 2


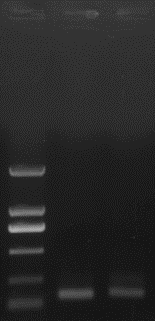

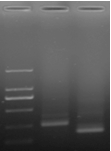

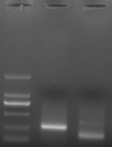


**2000bp**

**1000bp**

**750bp**

**500bp**

**250bp**

**100bp**

**2000bp**

**1000bp**

**750bp**

**500bp**

**250bp**

**100bp**

**2000bp**

**1000bp**

**750bp**

**500bp**

**250bp**

**100bp**

Figure S2 B: Fragments of RLM-5’ RACE (RNA Ligase Mediated 5’-RACE) (M, marker DL2000; 1, Bra016891 (SPL9); 2, Bra014599 (SPL15); 3, Bra003305 (SPL15), product of 2^nd^ nest-PCR; 4, Bra003305 (SPL15), product of 3^rd^ nest-PCR; 5, Bra004674 (SPL9), product of 2^nd^ nest-PCR; 6, Bra004674 (SPL9), product of 3^rd^ nest-PCR)


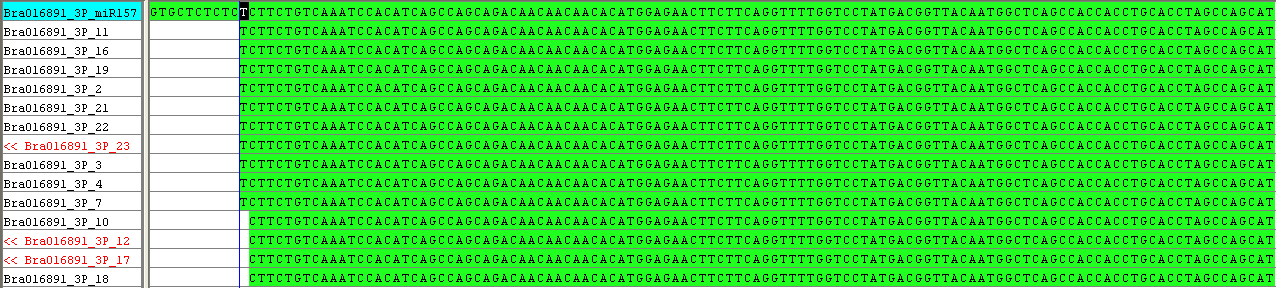


Figure S2 C: Blast analysis of sequencing results with target Bra016891 (SPL9) sequence


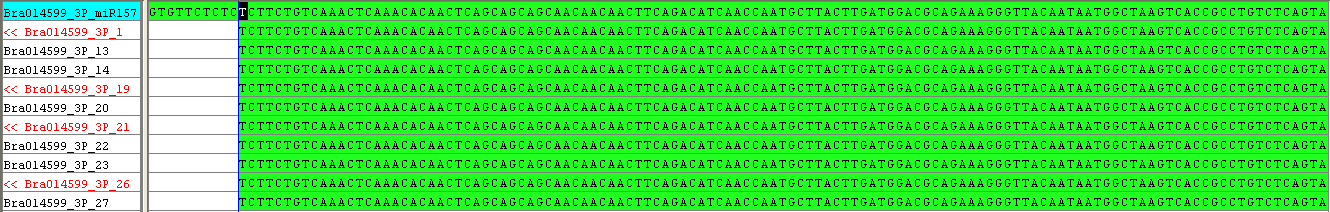


Figure S2 D: Blast analysis of sequencing results with target Bra014599 (SPL15) sequence


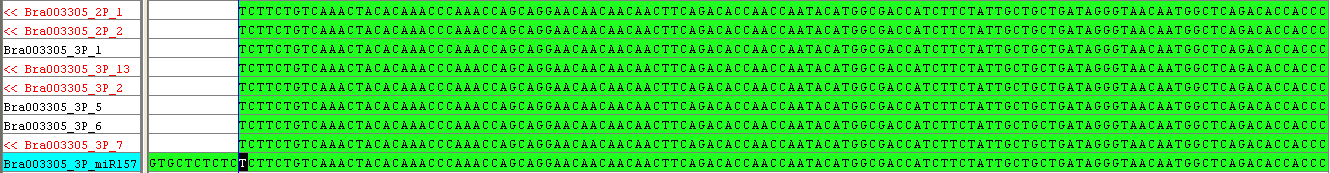


Figure S2 E: Blast analysis of sequencing results with target Bra003305 (SPL15) sequence


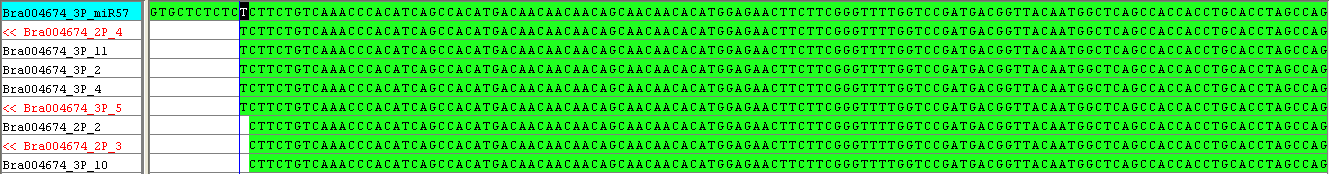


Figure S2 F: Blast analysis of sequencing results with target Bra004674 (SPL9) sequence
