# Supplementary material for: Exploring miRNAs involved in blue/UV-A light response in Brassica rapa reveals special regulatory mode during seedling development
Source: BMC Plant Biol. 2016 May 10;16:111. doi: 10.1186/s12870-016-0799-z (PMC4862165; doi:10.1186/s12870-016-0799-z)
Supplement: Additional file 8: Table S6. — Real-time RT-PCR examined miRNAs and primers. (DOC 35 kb) [file 12870_2016_799_MOESM8_ESM.doc]

**Table S6** Real-time reverse transcription-PCR examined miRNAs and primers

| miRNA ID | Sequence (5’-3’) | Reverse transcription primer | Forward primer | Amplification length (nt) |
| --- | --- | --- | --- | --- |
| BrmiR159 | UUUGGAUUGAAGGGAGCUCUA | GTCGTATCCAGTGCAGGGTCCGAGGTATTCGCACTGGATACGACTAGAGC | CGCCAGTTTGGATTGAAGGGA | 64 |
| BrmiRC0191 | UUGCAAGCCAGACAUUUCCUUU | GTCGTATCCAGTGCAGGGTCCGAGGTATTCGCACTGGATACGACAAAGGA | GACGCATTGCAAGCCAGACATT | 65 |
| BrmiRC0460 | UAGCUCCAGACUCAUUCACUCA | GTCGTATCCAGTGCAGGGTCCGAGGTATTCGCACTGGATACGACTGAGTG | CACGCATAGCTCCAGACTCATT | 65 |
| BrmiRC0323 | AUAAAUCCCAAGCAUCAUCCA | GTCGTATCCAGTGCAGGGTCCGAGGTATTCGCACTGGATACGACTGGATG | CACGCCATAAATCCCAAGCAT | 64 |
| BrmiRC0418 | CUUUGUCUAUCGUUUGGAAAAG | GTCGTATCCAGTGCAGGGTCCGAGGTATTCGCACTGGATACGACCTTTTC | CGAGCCCTTTGTCTATCGTTTG | 65 |
| BrmiRC0005 | CCCGCCUUGCAUCAACUGAAU | GTCGTATCCAGTGCAGGGTCCGAGGTATTCGCACTGGATACGACATTCAG | AGACTTCCCGCCTTGCATCAA | 64 |
| BrmiR157 | UUGACAGAAGAUAGAGAGCAC | GTCGTATCCAGTGCAGGGTCCGAGGTATTCGCACTGGATACGACGTGCTC | CGCCGACTTGACAGAAGATAGA | 65 |
| BrUBQ | Bra029570 | TTGTAGTCAGCCAAGGTACGACCA | TGGAGAGTTCCGACACCATTGACA | 130 |

Reverse primer: 5′-CCA GTG CAG GGT CCG AGG TA-3′
