# Supplementary material for: Exploring miRNAs involved in blue/UV-A light response in Brassica rapa reveals special regulatory mode during seedling development
Source: BMC Plant Biol. 2016 May 10;16:111. doi: 10.1186/s12870-016-0799-z (PMC4862165; doi:10.1186/s12870-016-0799-z)
Supplement: Additional file 9: Table S7. — Real-time PCR primers for detected genes. (DOC 32 kb) [file 12870_2016_799_MOESM9_ESM.doc]

**Table S7** Real-time PCR primers for detected genes

| Target Gene ID | Forward primer (5’-3’) | Reverse primer (5’-3’) | Amplification length (nt) |
| --- | --- | --- | --- |
| Bra004674(SPL9) | GGGAGGATCACTCCTTCTCTAT | GCCGTCTCATCACTCTTGTATC | 207 |
| Bra003305(SPL15) | GTTGTAGAAGACGGCTCGCT | CCCAAAACGCTTCTAATGGCA | 121 |
| Bra010949(SPL10) | AAAGTCAGTAGCGGCTCTC | CACGTTGCTGCAAGAATCTC | 129 |
| Bra038324(SPL6) | GTCACAACGAGCGAAGAAGA | CGTACAAGAAGCTACCAGGAAA | 100 |
| Bra027478(SPL2) | TTGCTCTTATCCATCCAAGGAC | CAGGTTGCTGTATTAACCCTCT | 87 |
| Bra030041(SPL10) | AGCTCTCCAGGTGGTATCT | CAACAGATGGAGGAGTCAAGAG | 146 |
| Bra033671(SPL2) | TCATTGCAGTGGGAGTGGGAT | GCTGGACGAGTAAAAAGTGGTGC | 126 |
| Bra022766(SPL13) | ACACTCCTCTTCTTCCTCCT | CTTGTCCTCTTGGAGCTTCTT | 114 |
| BrUBQ | TTGTAGTCAGCCAAGGTACGACCA | TGGAGAGTTCCGACACCATTGACA | 130 |
