# Supplementary material for: Exploring miRNAs involved in blue/UV-A light response in Brassica rapa reveals special regulatory mode during seedling development
Source: BMC Plant Biol. 2016 May 10;16:111. doi: 10.1186/s12870-016-0799-z (PMC4862165; doi:10.1186/s12870-016-0799-z)
Supplement: Additional file 10: Table S8. — Nest primers used in 5′-RACE PCR. (DOC 32 kb) [file 12870_2016_799_MOESM10_ESM.doc]

**Table S8** Nest primers used in 5’-RACE PCR

| Gene ID | Primer name | Sequence (5’-3’) |
| --- | --- | --- |
| Bra004674 | Br-SPL9-RLM NP1 | GCCATAAGCCCTTGTGTTCTC |
|  | Br-SPL9-RLM NP2 | AGTCGTTCCACCGCTTATCT |
|  | Br-SPL9-RLM NP3 | TACTGATGCTGCTGGCTAGGT |
| Bra016891 | Br-SPL9-RLM NP1 | TACTGCCTCCTATTTTGATGTTG |
|  | Br-SPL9-RLM NP2 | CTCACCCATTGTCGTATCTGTCTC |
|  | Br-SPL9-RLM NP3 | ATGCTGGCTAGGTGCAGGTGG |
| Bra003305 | Br-SPL15-RLM NP1 | GAGACCAGTTGAAATGCTGAGGGGA |
|  | Br-SPL15-RLM NP2 | GCTCAGAGATTTGGCTCAGTCCC |
|  | Br-SPL15-RLM NP3 | TTGATTCTGGACTGAAACGGGTGG |

5’ RACE Outer Primer：5’-CATGGCTACATGCTGACAGCCTA-3’

5’ RACE Inner Primer：5’-CGCGGATCCACAGCCTACTGATGATCAGTCGATG-3’
